# Characterizing Kinetics and Avidity of SARS-CoV-2 Antibody Responses in COVID-19 Greek Patients

**DOI:** 10.3390/v14040758

**Published:** 2022-04-05

**Authors:** Stavroula Labropoulou, Niki Vassilaki, Raphaela S. Milona, Evangelos Terpos, Marianna Politou, Vasiliki Pappa, Maria Pagoni, Elisavet Grouzi, Meletios A. Dimopoulos, Andreas Mentis, Mary Emmanouil, Emmanouil Angelakis

**Affiliations:** 1 Diagnostics Department and Public Health Laboratories, Hellenic Pasteur Institute, 11521 Athens, Greece; vlabropoulou@pasteur.gr (S.L.); mentis@pasteur.gr (A.M.); emmanouilm@pasteur.gr (M.E.); e.angelakis@pasteur.gr (E.A.); 2 Laboratory of Molecular Virology, Hellenic Pasteur Institute, 127 Vasilissis Sofias Avenue, 11521 Athens, Greece; raphaelasmilona@gmail.com; 3 Department of Clinical Therapeutics, School of Medicine, National and Kapodistrian University of Athens, 11528 Athens, Greece; eterpos@med.uoa.gr (E.T.); mdimop@med.uoa.gr (M.A.D.); 4 Hematology Laboratory Blood Bank, School of Medicine, Aretaieion Hospital, National and Kapodistrian University of Athens, 11528 Athens, Greece; mpolitou@med.uoa.gr; 5 Hematology Unit, Second Department of Internal Medicine, School of Medicine, Attikon University General Hospital, National and Kapodistrian University of Athens, 12461 Athens, Greece; vas_pappa@yahoo.com; 6 BMT Unit, Department of Hematology and Lymphomas, Evangelismos General Hospital, 10676 Athens, Greece; marianpagoni@yahoo.com; 7 Department of Transfusion Service and Clinical Hemostasis, “Saint Savvas” Oncology Hospital, 11522 Athens, Greece; egrouzi@otenet.gr; 8 IHU-Méditerranée Infection, Aix-Marseille University, 19-21 Boulevard Jean Moulin, 13005 Marseille, France

**Keywords:** SARS-CoV-2, COVID-19, IgG kinetics, antibody avidity

## Abstract

In-depth understanding of the immune response provoked by SARS-CoV-2 infection is necessary, as there is a great risk of reinfection and a difficulty in achieving herd immunity due to a decline in both antibody concentration and avidity. Avidity testing, however, could overcome variability in the immune response associated with sex or clinical symptoms, and thus differentiate between recent and past infections. In this context, here, we analyzed SARS-CoV-2 antibody kinetics and avidity in Greek hospitalized (26%) and non-hospitalized (74%) COVID-19 patients (N = 71) in the course of up to 15 months after their infection to improve the accuracy of the serological diagnosis in dating the onset of the infection. The results showed that IgG-S1 levels decline significantly at four months (*p* = 0.0239) in both groups of patients and are higher in hospitalized ones (up to 2.1-fold, *p* < 0.001). Additionally, hospitalized patients’ titers drop greatly and are equalized to non-hospitalized ones only at a time-point of twelve to fifteen months. Antibody levels of women in total remain more stable months after infection, compared to men. Furthermore, we examined the differential maturation of IgG avidity after SARS-CoV-2 infection, showing an incomplete maturation of avidity that results in a plateau at four months after infection. We also defined 38.2% avidity (sensitivity: 58.9%, specificity: 90.91%) as an appropriate “cut-off” that could be used to determine the stage of infection before avidity reaches a plateau.

## 1. Introduction

In 2020, the World Health Organization (WHO) characterized COVID-19 as a pandemic, which greatly impacted health care and socio-economic systems all over the globe [1,2]. SARS-CoV-2 causes a wide range of symptoms, such as fever, fatigue, dry cough, headaches and dyspnea, and more severe manifestations such as acute pneumonia and neurological complications [3], including damage in the dopaminergic system [4,5]. Severe deterioration in some cases has been associated with a dysregulated immune state and hyperinflammation [6,7]. Managing this pandemic and preventing further transmission of SARS-CoV-2 requires more than diagnosing, treating, and putting symptomatic patients in quarantine. It is of equal importance that serological tests are developed to define asymptomatic and infected individuals, seronegative people, and people with positive immune responses [8]. So far, rapid SARS-CoV-2 antigen detection tests and real time reverse transcription-polymerase chain reaction (RT-PCR) assays are those widely used for efficient detection of infected individuals in laboratories [9]. However, they do not provide all the information needed.

SARS-CoV-2 antibody kinetics and avidity (functional affinity) assays are far more useful on the overall surveillance of the pandemic [8,10]. COVID-19 patients typically produce detectable anti-SARS antibodies within several weeks post infection [11,12]. Antibody kinetics research has shown that within the first months of infection antibodies are produced and degraded rapidly, but after a time period the degradation rhythm is much slower [13,14]. In addition, it has been indicated that anti-SARS antibodies persist for over a year [15,16,17]. Because IgG antibodies show a great correlation with anti-S neutralizing antibody titers and decay long after IgMs and IgAs, they are of greater value in epidemiological studies [18,19]. As a result, many studies have tried to investigate IgG levels association with a variety of factors, discovering weak or no correlation with sex [20,21,22,23,24], ambiguous results regarding correlation with age [20,21,25,26,27], and higher antibody levels in accordance with disease severity [12,28].

Antibody avidity against SARS-CoV-2 in COVID-19 patients, unlike the pattern of immune responses against other viruses, is low even months after infection [29,30,31,32]. Avidity testing has been used in diagnosis of recent infections in many viruses, including Epstein-Barr virus [33], HIV [34], West Nile Virus [35], and other SARS-CoV infections, as a system that can resolve the problem of the serological response’s high variability in those infections [36]. Although this incomplete avidity maturation of IgGs targeting SARS-CoV-2 raises concerns about the efficiency of this method in determining the stage of the infection in COVID-19 patients, it seems that with the right selection of “cut-off” values, avidity testing could be helpful in achieving that goal [37]. In this context, we analyzed SARS-CoV-2 seroprevalence and antibody kinetics over a one year period, and furthermore we developed an avidity test to improve the accuracy of the serological diagnosis in dating the onset of the infection and to distinguish past from recent SARS-CoV-2 infections.

## 2. Materials and Methods

### 2.1. Patients

Blood samples were collected in a 5 mL vaccutainer from hospitalized and non-hospitalized COVID-19 patients in 2 or 3 time-points over a period of 1 year (up to 15 months), during their infection and after recovery. All patients had confirmed SARS-CoV-2 infection via a positive RNA nasopharyngeal swab PCR test, the time of which was considered as month zero (T0), and a known date of symptom onset. In total, the 71 patients contributed 253 serum samples. The serum samples were separated after centrifugation at 3000 rpm for 5 min.

### 2.2. Serological Assays

Anti-SARS-CoV-2 IgG enzyme-linked immunosorbent assay (ELISA) for the S1 domain of Spike protein (EUROIMMUN Medizinische Labordiagnostika AG, Germany, Lübeck) was performed according to the manufacturers’ protocols. Optical density (OD) of the sample divided by calibrator provided index values ratio for which ≥1.1 was considered positive and ≥0.8–1.1 was considered indeterminate.

Avidity assays were performed for samples that had Euroimmun index values ratios ≥ 1.1 (i.e., seropositive specimens). Euroimmun anti-SARS-CoV-2 ELISA IgG kits (EUROIMMUN Medizinische Labordiagnostika AG, Germany, Lübeck) were used with modified protocols for avidity testing. Each reaction utilized the following components: 100 µL of diluted plasma (1:101 dilution) and 100 µL of undiluted positive, negative, or calibrator controls. Plates containing reaction components were incubated for 1 h at 37 °C followed by 1 wash. Urea 5M (Sigma Aldrich Chemie GmbH, Taufkirchen, Germany), 300 µL, diluted in the appropriate wash buffer was added to the plates and incubated at RT for 10 min. The specific concentration of urea was selected based on the range of IgG titers (index values) determined in the present experiments and the previous report of Benner et al. [30], where a similar experimental setup was used to measure IgG avidity. Plates were washed 3 times followed by manufacturer’s protocol for addition of conjugate and substrate. Ratios of sample with urea concentration to sample without urea (either AU or ODn) were used for calculation of percentage: OD (sample with urea)/OD (sample without urea) × 100%.

### 2.3. Statistical Analyses

At first, descriptive statistics were calculated. Based on the D’Agostino and Pearson test, replaced by the Shapiro–Wilk test in smaller sample sizes, we verified if data distribution was parametric or non-parametric and subsequently selected the appropriate statistical test for analysis. Analyses of anti-SARS-CoV-2 S1 IgG levels and of antibody avidity levels in relation to time (four unpaired groups) were performed using Kruskal–Wallis test. Dunn’s correction was used for post hoc analysis of two time-points at a time. Comparison of IgG titers between groups (based on their sex or clinical features) was performed using Student’s *t*-test (unpaired) for normal distributions, or Mann–Whitney U test for non-normal distribution. Fisher’s exact test was used to confirm that the ratio of women/men does not change significantly between hospitalized and non-hospitalized patients and vice versa. In addition, we estimated the statistical significance of the reduction in antibody levels over time within each group and between groups, using repeated measures one-way ANOVA and repeated measures two-way ANOVA, respectively, with the Geisser–Greenhouse correction, as suggested [38]. In the case of two time-points comparison at a time, we used post hoc analysis with Tukey’s correction (as an integral part of one-way ANOVA). Lastly, we evaluated the diagnostic accuracy of antibody avidity as a biomarker that distinguishes amongst acute and past infections with the receiver operating characteristic (ROC) regression analysis and calculated for each time-point used the area under the ROC curve (AUC), as well as the cut-off value based on Youden’s index. All statistical analyses were performed using GraphPad (GraphPad Prism version 9.0.0 free trial, GraphPad Software Inc., San Diego, CA, USA) and *p* < 0.05 (two-tailed) was considered statistically significant.

## 3. Results

### 3.1. Characteristics of the Study Population

Overall, we analyzed samples from 71 patients (males 62%) (Table 1). Regarding their clinical features, data were provided for 66 of them (93%), while for 5 of them (7%) there were no data provided. Out of those, 17 were hospitalized (26%) in severe or critical condition during their infection, while 49 were not hospitalized (74%), showing mild COVID-19 clinical symptoms, including cough, sore throat, mild fever below 38 °C, and loss of smell. Sample collection was conducted at various time-points up to fifteen months after patients’ first positive PCR test (T0) and the dates of the sample collection were grouped into four categories: 1–3 months, 4–5 months, 6–8 months, and 12–15 months. Sampling occurred two (24%) or three times (76%) for each patient. Patients whose last time-point was between six to eight months were analyzed separately from those whose last time-point is twelve to fifteen months. Anti-SARS-CoV-2 S1 IgGs were detected, quantified, and analyzed in the samples in order to determine antibody kinetics and their correlation with sex and clinical features. All patients were seropositive for anti-Spike IgG at early time-points (≤5 months) based on the cut-off of the assay (≥1.1 index values). Only seven measurements in total were lower than the threshold but they were from samples collected over six months after infection. In addition, for 40 patients (56%), antibody avidity was also measured in samples that were seropositive in order to determine cut off values that differentiate recent from older infections.

### 3.2. Anti-SARS-CoV-2 S1 Antibody Kinetics

**Figure 1:**
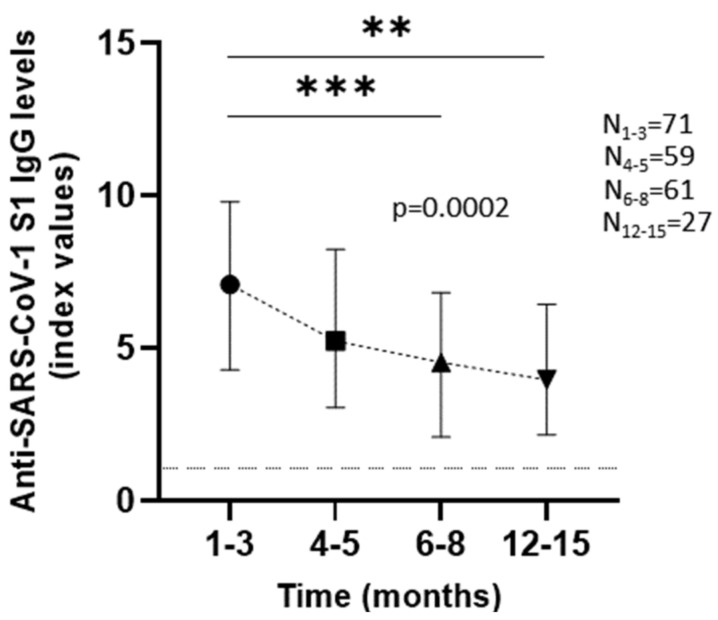
Anti-S1 IgGs of all patients included in the study (hospitalized or not) were measured, using Euroimmune anti-SARS-CoV-2 ELISA IgG assay, at several time-points up to fifteen months. Points at the graph represent the medians of IgG concentration at each time-point and error bars indicate the interquartile range (IQR). Horizontal dashed line represents the cut-off value of the assay used. *p* value indicated on the graph was calculated using Kruskal–Wallis test for all time-points collectively, while post hoc analysis of two time-points at a time also revealed statistical significance, with *** *p* < 0.001 and ** *p* < 0.01.

**Figure 2:**
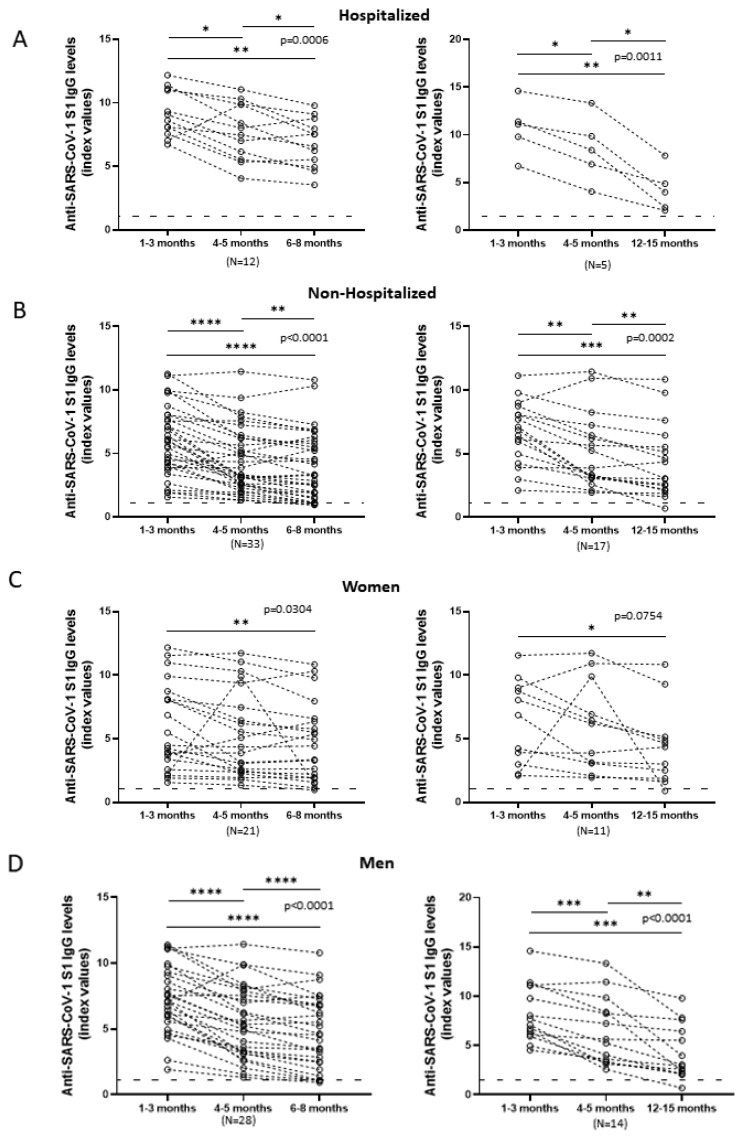
IgG responses targeting SARS-CoV-2 S1 over time for all different groups analyzed. Anti-S1 IgGs of all patients included in the study were measured at various time-points up to fifteen months. Patients whose last sample collection occurred six to eight months after infection (left side of the figure) were analyzed separately from those whose last sample collection occurred twelve to fifteen months after infection (right side of the panel). Circles on the graph represent a measurement at a certain time-point while dashed lines connect the three measurements of a single patient. Horizontal dashed lines represent the cut-off value of the assay used. *p* values indicated on the graph were calculated using repeated measures one-way ANOVA for all time-points, while post hoc analyses of two time-points at a time revealed statistical significance with * *p* < 0.05, ** *p* < 0.01, *** *p* < 0.001 and **** *p* < 0.0001. Antibody levels reduction of (**A**): All hospitalized patients. (**B**): All non-hospitalized patients. (**C**): All female patients. (**D**): All male patients.

Anti-SARS-CoV-2 S1 IgG titers of all patients displayed a drop within the first months of the patient’s recovery (*p* = 0.0002). The median of the antibody levels in the first one to three months was 7.1 index values (IQR = 4.3–9.8) and decreased to a median of 5.24 index values (IQR = 3.06–8.24) at four to five months, 4.55 index values (IQR = 2.09–6.83) at six to eight months, and 3.97 index values (IQR = 2.17–6.44) at twelve to fifteen months after infection (Figure 1). Statistical analysis of two time-points at a time showed that, at the first time-point (1–3 months), IgG titers were significantly higher than those at later time-points (*p* = 0.0005 for 6–8 months and *p* = 0.0046 for 12–15 months). However, the decrease amongst the later time-points was not statistically significant. That could indicate that the antibody levels could drop at first, as we detect a significant 0.4-fold reduction (1–3 months vs. 6–8 months), but remain at certain levels for a period of time after that. Analysis of each individual group (Figure 2) confirmed statistically significant antibody decrease over time regardless of sex or clinical features. Only one group sidetracked from that pattern, women whose third time-point was twelve to fifteen months (*p* = 0.0754) (Figure 2C right side of the panel), but statistical analysis of the time-points at a time showed a significant decrease between extreme time-points (1–3 months vs. 12–15 months), considering the small number of patients analyzed at this specific graph that could also be attributed to random factors.

### 3.3. Comparison of Anti-SARS-CoV-2 S1 Antibody Kinetics among Hospitalized and Non-Hospitalized Patients

**Figure 3:**
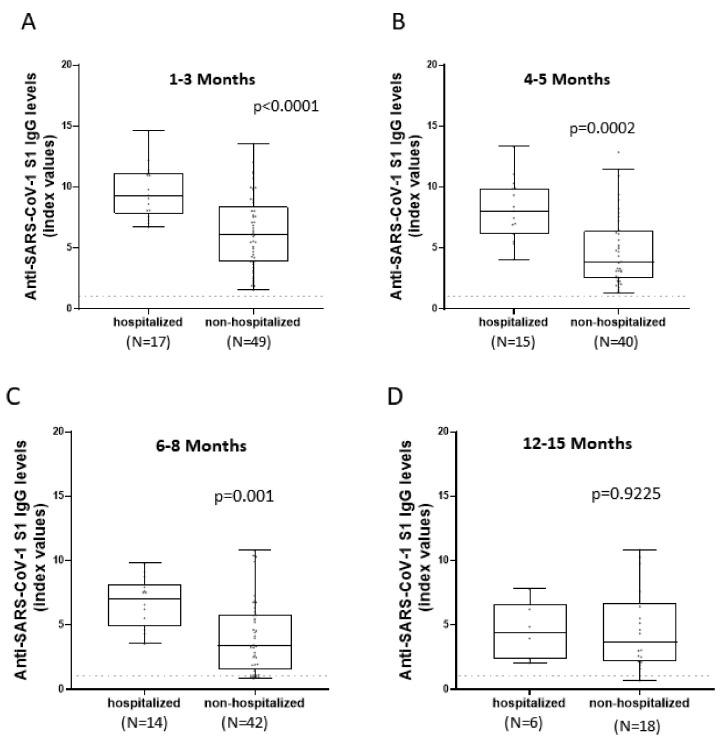
Comparison of IgG responses targeting SARS-CoV-2 S1 between hospitalized and non-hospitalized patients in various time-points. Hospitalized and non-hospitalized IgG responses were measured at various time-points as different groups. Statistical analysis occurred between hospitalized and non-hospitalized patients, regardless of their sex, and Unpaired *t*-test (**A**) and Mann–Whitney test (**B**–**D**) were used to determine whether there was a significant difference between the antibody titers of the two groups at a certain time-point each time. Horizontal dashed lines represent the cut-off value of the assay used. Comparison of anti-SARS-CoV-2 IgG titers between hospitalized and non-hospitalized patients (**A**): In samples collected one to three months after infection. (**B**): In samples collected four to five months after infection. (**C**): In samples collected six to eight months after infection. (**D**): In samples collected twelve to fifteen months after infection.

Both hospitalized and non-hospitalized patients displayed a reduction in IgG concentration over time (Figure 3). It is, however, evident that the mean of antibody titers of hospitalized patients was higher than non-hospitalized up to eight months after infection. Samples collected one to three months after infection were analyzed using unpaired *t*-test as they follow a Gaussian distribution, and their means were statistically different (*p* < 0.0001). Specifically, hospitalized patients had a mean of 9.64 index values (SD = 2.14) and a median of 9.32 index values (IQR = 7.82–11.15) and were 1.5-folds higher than non-hospitalized ones which had a mean of 6.258 index values (SD = 3.01) and median of 6.07 index values (IQR = 3.88–8.38). At all other time-points, Mann–Whitney test was used as the samples did not follow a normal distribution. As a result, we could not compare means. At four to five months, the median of IgG titers for hospitalized patients was 8.03 index values (IQR = 6.16–9.9) and presented a 2.1-fold increase in comparison to non-hospitalized patients’ median, 3.84 index values (IQR = 2.52–6.4). Likewise, at six to eight months there was a 2.1-fold increase of hospitalized antibody levels, (median = 7.06 index values; IQR = 4.86–8.15) compared to non-hospitalized individuals (median = 3.4 index values; IQR = 1.54–5.84). The differences up to this point were statistically different (*p* = 0.0002 and *p* = 0.001, respectively). However, for samples collected twelve to fifteen months after infection there was no statistical difference between the distributions of the two groups (*p* = 0.9225) and their medians, 4.42 index values (IQR = 2.31–6.62) for hospitalized patients and 3.7 index values (IQR = 2.16–6.74) for non-hospitalized ones, were similar.

### 3.4. Comparison of Anti-SARS-CoV-2 S1 Antibody Kinetics among Female and Male Patients

**Figure 4:**
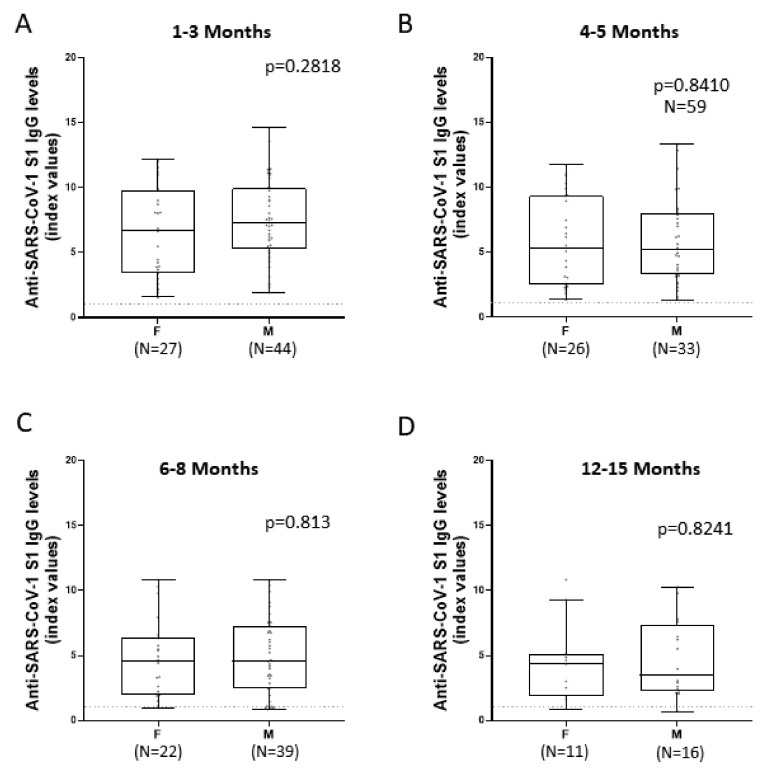
Comparison of IgG responses targeting SARS-CoV-2 S1 between female and male patients in various time-points. IgG responses were measured at various time-points and grouped into two categories, male and female. Statistical analysis occurred between female and male patients, regardless of the severity of their disease, and Mann–Whitney test (**A**,**B**) and Unpaired *t* test (**C**,**D**) were used to determine whether there was a significant difference between the antibody titers of the two groups at a certain time-point each time. Horizontal dashed lines represent the cut-off value of the assay used. Comparison of anti-SARS-CoV-2 IgG titers between female and male patients (**A**): In samples collected one to three months after infection. (**B**): In samples collected four to five months after infection. (**C**): In samples collected six to eight months after infection. (**D**): In sample collected twelve to fifteen months after infection.

IgG titers against SARS-CoV-2 S1 did not seem to differentiate significantly depending on the patient’s sex (Figure 4). Mann–Whitney test was used to statistically analyze antibody concentration for the first two time-points (1–3 months and 4–5 months) because the data were not drawn from a normal distribution. For the other two time-points (6–8 months and 12–15 months), unpaired *t*-test was used for the statistical analysis of the samples because the data are normally distributed. *p* values were 0.2818, 0.841, 0.813, and 0.8241, respectively and they were in all four cases not statistically important.

### 3.5. Comparison of Anti-SARS-CoV-2 S1 Antibody Kinetics among Hospitalized and Non-Hospitalized and Female and Male Patients in Relation to Time

**Figure 5:**
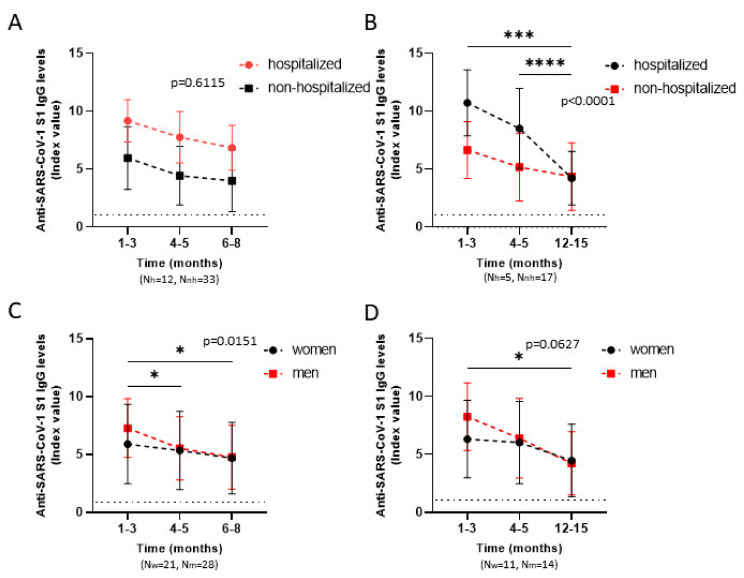
Comparison of IgG responses targeting SARS-CoV-2 S1 between female and male patients and between hospitalized and non-hospitalized patients in relation to time. IgG responses were measured at various time-points and grouped into categories regarding their sex and their clinical features. Patients whose last sample was collected six to eight months or twelve to fifteen months after infection were analyzed separately. Repeated measures two-way ANOVA with the Geisser–Greenhouse correction was performed to determine whether the difference between the groups analyzed (hospitalized vs. non-hospitalized and men vs. women) is identical: (a) among all time-points tested and the *p* values extracted from this analysis are displayed on the panels, or (b) between two time-points at a time and the respective *p* values extracted are * *p* < 0.05, *** *p* < 0.001, and **** *p* < 0.0001. Horizontal dashed lines represent the cut-off value of the assay used. Comparison of anti-SARS-CoV-2 IgG titers between hospitalized and non-hospitalized individuals, (**A**): whose last samples is collected six to eight months after infection. (**B**): whose last samples is collected twelve to fifteen months after infection. Between female and male patients, (**C**): whose last samples is collected six to eight months after infection. (**D**): whose last samples is collected twelve to fifteen months after infection.

Comparison of hospitalized and non-hospitalized patients, up to eight months, indicates that hospitalized patients have higher antibody titers, but they decrease in a rhythm similar to that of non-hospitalized individuals, and as a result their difference remains similar (*p* = 0.6115) (Figure 5A). However, that pattern was not evident when analyzing later time-points. At samples collected at twelve to fifteen months, IgG titers of hospitalized patients decreased significantly and were almost equalized to those of non-hospitalized individuals (*p* < 0.0001) (Figure 5B). This rapid decrease in antibody titers appeared after the first four to five months. On the contrary, comparison of female and male patients, suggests that men had higher antibody levels at one to three months after infection but decreased significantly over time (*p* = 0.0151) (Figure 5C). Although there was only a significant difference in the way antibody levels drop from 1–3 to 4–5 months in one data analysis (Figure 5C), it appears safe to assume that at least after six months, men’s IgG titers have decreased significantly and are similar to women’s titers, which may be slightly lower at first but drop rather smoothly.

### 3.6. Antibody Avidity Maturation over Time Is an Important Tool in Determining Stage of the Infection

**Figure 6:**
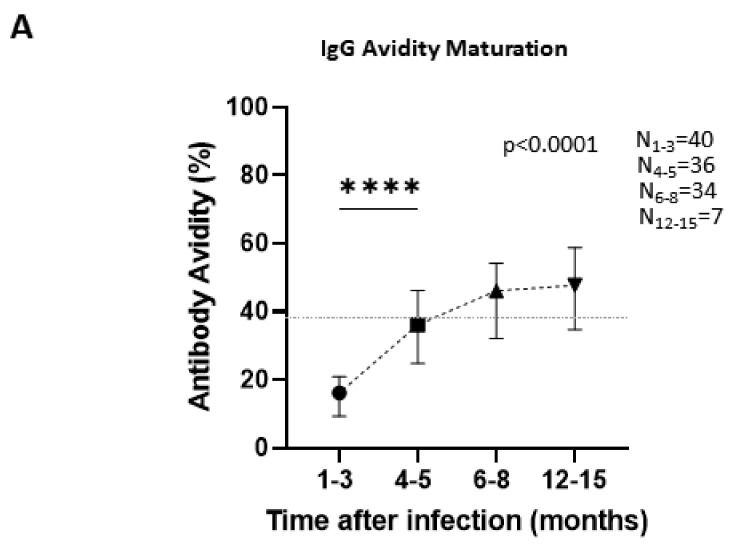

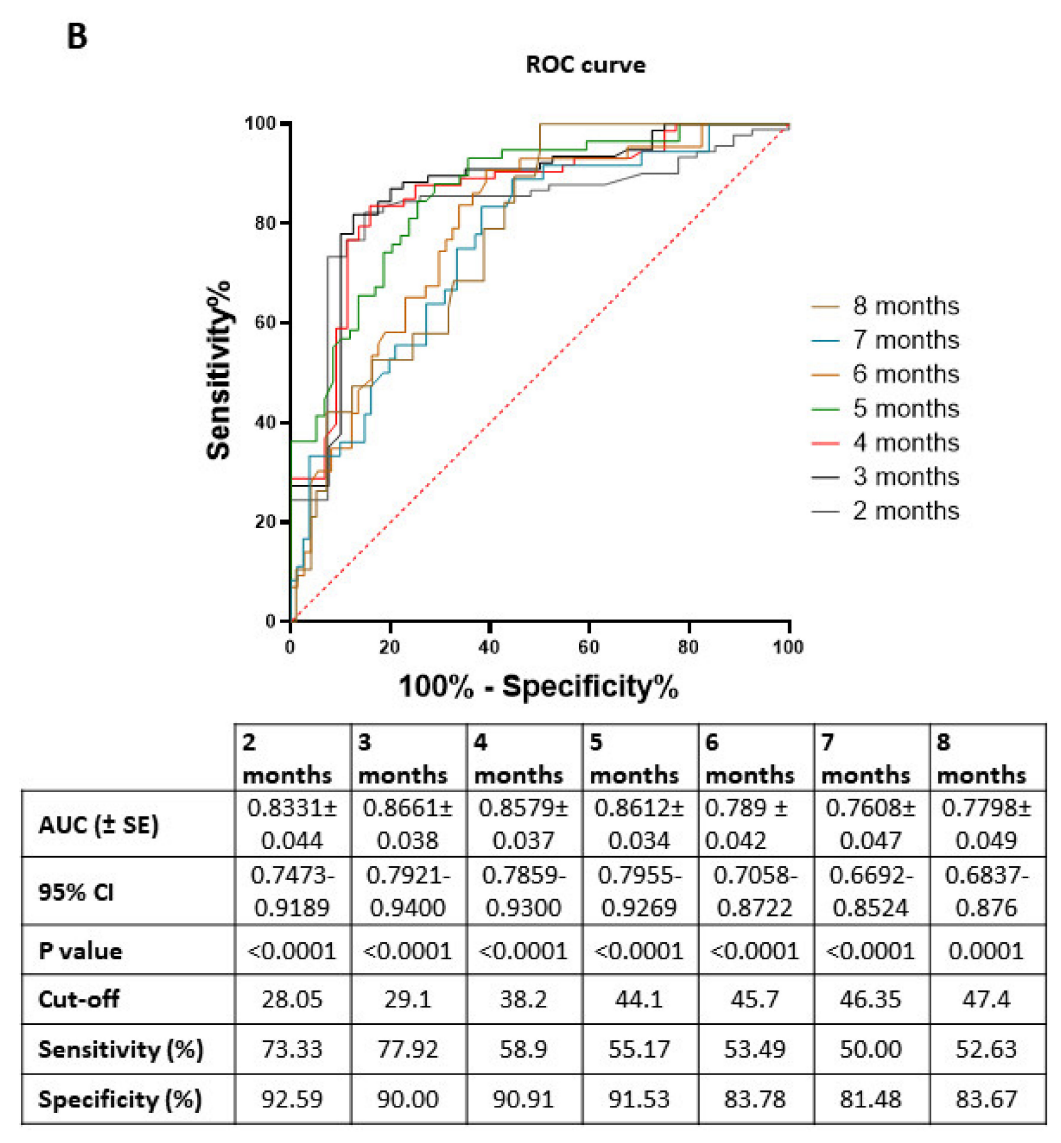
Antibody avidity percentages in COVID-19 patients over time after infection and classification of time since previous infection. (**A**): Antibody avidity of all patients included in the study (hospitalized or not) were measured at several time-points up to fifteen months. Points at the graph represent the median of avidities at each time-point and error bars indicate the interquartile range (IQR). Horizontal dashed line represents the cut-off value of antibody avidity that has been suggested to differentiate between infections that occurred three or more than three months before sampling. *p* value indicated on the graph was calculated using Kruskal–Wallis test for all time-points collectively and post hoc analysis of two time-points at a time revealed statistical significance with **** *p* < 0.0001. (**B**): ROC curves were created to identify the time-point discriminating between recent and past infection, by comparing avidity values of samples collected before and after two (grey line), three (black line), four (red line), five (green line), six (orange line), seven (blue line), and eight months (brown line). Area under the curve for each ROC curve quantifies the overall ability of the test to discriminate between those individuals that were infected before or after the time-point we used as a distinguishing factor. Cut-offs for each ROC curve that exhibit the best combination of sensitivity and specificity were selected.

Avidity of antibodies produced against SARS-CoV-2 increases over time as a result of IgG maturation in order to better target viral epitopes. In one to three months after infection, antibody avidity had a median of 16.1% (IQR = 9.32–20.9%) and was increased at four to five months to a median of 36.1% (IQR = 24.8–46.23%), at six to eight months to a median of 46.1% (IQR = 32.13–54.13%), and at twelve to fifteen months to a median of 47.7% (IQR = 34.7–58.8%) (Figure 6A). Although this gradual increase is statistically important (*p* < 0.0001), when comparing IgG levels of two neighboring time-points at a time, we observed that the first time-point (1–3 months) showed statistically significant difference as compared to the second one (4–5 months) (*p* < 0.0001) but there is no statistical significance between the second and the third time point (6–8 months) or between the third and the last time-point (12–15 months). Consequently, it seems that avidity maturation between four to fifteen months does not vary significantly, as it starts approaching a plateau at four months. To specify the exact time-point that can distinguish recent from past infections, we performed a measurement of antibody avidity and ROC curve analysis. Diagnosing infections that occurred before or after two or three months required using 28.05% and 29.1% avidity as cut-offs, respectively, in order to ensure the best sensitivity and specificity combination based on Youden’s index. At four months, an avidity test with a cut-off of 38.2% had lower sensitivity percentages. At five, six, seven, and eight months, optimal cut-offs varied slightly from 44.1% to 47.4%. That is a an obvious and expected aftermath of the incomplete avidity maturation that keeps anti-SARS-CoV-2 antibody avidity at intermediate levels even several months after infection. Although all diagnostical algorithms presented had statistical value (*p* ≤ 0.0001), cut-offs at time-points prior to four months or afterwards four months were really close to one another. At four months, cut-off is quite far from its neighboring time-points and seems to be the optimum time-point before avidity reaches a definite plateau to be used for determining the stage of infection.

## 4. Discussion

Understanding antibody kinetics and avidity maturation is a top priority on SARS-CoV-2 research. We found that anti-SARS-CoV-2 S1 IgG titers have declined significantly at four months after infection but persist in lower concentrations for more than twelve months. Furthermore, we showed that avidity maturation approaches a plateau four months after infection at intermediate levels that do not surpass 60% for most patients. This decrease in antibody concentration, as well as incomplete avidity maturation that keeps avidity levels at low percentages even up to fifteen months, is believed to increase the risk of reinfection, and negatively affect vaccine efficacy. Several studies have indicated that antibody avidity against SARS-CoV-2, unlike the pattern of immune responses against other viruses, is low even months after infection [29,30,31,32]. Although incomplete avidity maturation of IgGs targeting SARS-CoV-2 keeps avidity levels low, it seems that with the right selection of “cut-off” values, avidity testing could be helpful in determining the stage of the infection in COVID-19 patients [37]. Our data are in agreement with previous studies that show a decrease in antibody levels several months (3–13.5 months) after infection [17,23,29,39,40], as well as an increase in antibody avidity that reaches a plateau a few months (21 days–8 months) after infection [29,30,31,32,37,41,42,43]. An added value of the present study is that data have been obtained during a longer time course, up to fifteen months, as compared to previous reports, where antibody avidity was addressed at time-points up to eight months after infection. Differences in avidity percentages among the published studies could be attributed to different concentrations of urea used [30,31,38]. Indeed, higher concentrations of urea result in lower avidity percentages for a given time-point. Similar data to the ones presented here were obtained for concentrations of urea close to the one used in the present study [43]. At any case, long-term studies conclude that, in SARS-CoV-2 infection, avidity maturation is incomplete. Except for the time that has passed since the infection, sex, age, genetic variations, and disease severity are also proven to be strong predictors of the immune response to many human pathogens and the vaccines against them, including SARS-CoV-2 [44,45,46]. As a result, statistical analysis of their correlation could be a great tool for later pandemics’ management regarding the determination of high-risk individuals and the serological test best suited for each group [47].

Disease severity is one factor greatly associated with the immune response provoked by this viral infection [28,48,49]. COVID-19 patients that were in need of hospitalization display higher levels of antibodies against SARS-CoV-2 for the first eight months after infection. At sample collected twelve to fifteen months after infection, however, their IgG titers have dropped significantly and are equalized to non-hospitalized patients’ titers. Possible explanations for this persistence of IgG titers in hospitalized individuals up to eight months after infection are that elevated inflammatory response and secondary antibody-mediated organ damage could result in a more robust immune response that declines, eventually, after the patient has long recovered [50,51].

Although male and female patients do not vary significantly at any time-point individually, in agreement with previous findings [48], we observe a statistically significant difference in the way their IgG concentrations decline. Interestingly, women’s antibody response is fairly stable over the months in comparison with men’s IgG production, which is slightly elevated at first and decreases considerably. However, this pattern is prominent only for the first months after infection. Later than four months post infection, both women’s and men’s antibody titers decrease in an analogous manner. This confusing pattern in immune responses amongst the sexes could be explained by studies that report higher severity and death rates of male COVID-19 patients [52,53,54], as disease severity could result in higher antibody concentrations at first and stable anti-SARS-CoV-2 IgG production could protect female patients from severe clinical symptoms [37].

On another note, antibody avidity testing has great value in distinguishing acute from past infection, because antibodies bind weakly to their epitopes at first, but their affinity increases over time. As a diagnostic tool, it has been excessively used in estimating time of infection in pregnant women [55]. In contrast to anti-SARS-CoV-2 S1 IgG concentration that decline over time, we observe an increase in antibody avidity at four months post infection. Time-dependent antibody maturation, which has been evident in other studies as well, has underlined its importance as a diagnostic test [8,56]. However, in order to utilize it diagnostically we need to determine at which time-point after infection avidity peaks and reaches a plateau. We have demonstrated, by statistical analysis of avidity percentages at various time-points and by building ROC curves, that avidity up to three months is low and increases significantly at four months, but later time-points do not differentiate significantly. Thus, we suggest that an avidity test with a cut off value of 38.2% could distinguish individuals infected within the previous four months (avidity lower than cut off) or more than four months before the sample collection (avidity higher than cut off), with 58.9% sensitivity and 90.91% specificity. This diagnostic algorithm, according to our analysis, constitutes an efficient test in diagnosing recent and past infections, with the time point of 4 months being the longest time point at which we can determine the stage of infection before avidity reaches a plateau.

Classification of COVID-19 patients’ antibody levels as an efficient diagnostic test to distinguish infections that occurred within the first months after infections or after these time-points has also been suggested [10]. This suggestion agrees with our and other researchers’ observations [29,37]—that the breakpoint of decreasing IgG titers correlates with the time-point of avidity stabilization due to incomplete avidity maturation. Antibody concentration remains at low but stable levels and avidity remains at intermediate but stable levels four to fifteen months after infection, thus estimating time of infection using later time-points as a distinguishing factor for acute or past infection would be extremely confusing.

SARS-CoV-2 IgG avidity test has been also emphasized as an indirect method of neutralizing activity measurement, based on previous studies correlating these two parameters by using tissue culture-based antibody neutralization assays [30,57]. However, in a different experimental setup, where anti-SARS-CoV-2 Spike receptor binding domain (RBD) neutralizing antibodies in patients’ sera were measured by ELISA, we detected no correlation with anti-S1 antibody avidity (Figure S1).

In conclusion, the present study constitutes the effort to clarify the kinetics and avidity of IgG antibodies against SARS-CoV-2 S1 in symptomatic patients with COVID-19 in Greece. Taking into consideration the small sample size, the obtained results highlight the importance of IgG antibody avidity as a laboratory tool in estimating the time-point after infection that could discriminate between recent and older infection. Thus, they can contribute to the surveillance of current and future pandemics. Furthermore, comparison of antibody avidity among patients infected with different SARS-CoV-2 variants of concern in future studies will complement current limited knowledge [57,58] on the association between virus genetic diversity and immune response.

## Figures and Tables

**Table 1:** Demographic data and anti-SARs-CoV-2 S1 IgG and Avidity titers of COVID-19 patients.

	**71** **(Total)**	**IgG Levels (Median; IQR)**			
		**1–3 Months** **(N = 71)**	**4–5 Months** **(N = 59)**	**6–8 Months** **(N = 61)**	**12–15 Months** **(N = 27)**
**Patients**		7.1 (4.3–9.8)	5.24 (3.06–8.24)	4.55 (2.09–6.83)	3.97 (2.17–6.44)
	40	**Antibody Avidity % (Median; IQR)**			
		**1–3 Months** **(N = 40)**	**4–5 Months** **(N = 36)**	**6–8 Months** **(N = 34)**	**12–15 Months** **(N = 7)**
		16.1 (9.32–20.9)	36.1 (24.8–46.23)	46.1 (32.13–54.13)	47.7 (34.7–58.8)
		**IgG levels (Median; IQR)**			
		**1–3 Months**	**4–5 Months**	**6–8 Months**	**12–15 Months**
**Sex**					
Male	44	7.21 (5.2–9.92)	5.24 (3.24–7.96)	4.55 (2.48–7.28)	3.51 (2.22–7.32)
Female	27	6.65 (3.38–9.8)	5.3 (2.47–9.35)	4.53 (1.94–6.41)	4.35 (1.88–5.15)
	*p* value > 0.999 ^a^				
**Clinical features**					
Hospitalized	17	9.32 (7.82–11.15)	8.03 (6.16–9.9)	7.06 (4.86–8.15)	4.42 (2.31–6.62)
Non-Hospitalized	49	6.07 (3.885–8.385)	3.84 (2.52–6.4)	3.4 (1.54–5.84)	3.7 (2.16–6.74)
NA	5				
	*p* value > 0.999 ^b^				

NR: Data not recorded; IQR: inter-quartile range; ^a,b^ Fisher’s exact test for the ratios: ^a^ No hospitalized/No non-hospitalized between male and female patients and ^b^ No females/No males between hospitalized and non-hospitalized patients.

## Data Availability

All relevant data are within the manuscript and its Supplementary Materials.

## Supplementary Materials

The following supporting information can be downloaded at: https://www.mdpi.com/article/10.3390/v14040758/s1. Figure S1, Anti-Spike RBD neutralizing antibody levels in COVID-19 patients over time after infection and correlation with antibody avidity.
