# Supplementary material for: Characterizing Kinetics and Avidity of SARS-CoV-2 Antibody Responses in COVID-19 Greek Patients
Source: Viruses. 2022 Apr 5;14(4):758. doi: 10.3390/v14040758 (PMC9024518; doi:10.3390/v14040758)
Supplement: Supplementary file 1 [file viruses-14-00758-s001.zip › viruses-1610150-supplementary.pdf]

viruses

Article

Characterizing Kinetics and Avidity of SARS-CoV-2 Antibody Responses in COVID-19 Greek Patients

Stavroula Labropoulou [1,†], Niki Vassilaki [2,*,†], Raphaela S. Milona [2], Evangelos Terpos [3], Marianna Politou [4], Vasiliki Pappa [5], Maria Pagoni [6], Elisavet Grouzi [7], Meletios A. Dimopoulos [3], Andreas Mentis [1], Mary Emmanouil [1] and Emmanouil Angelakis [1,8]

[1] Diagnostics Department and Public Health Laboratories, Hellenic Pasteur Institute, 11521 Athens, Greece; vlabropoulou@pasteur.gr (S.L.); mentis@pasteur.gr (A.M.); emmanouilm@pasteur.gr (M.E.); e.angelakis@pasteur.gr (E.A.)
[2] Laboratory of Molecular Virology, Hellenic Pasteur Institute, 127 Vasilissis Sofias Avenue, 11521 Athens, Greece; raphaelasmilona@gmail.com
[3] Department of Clinical Therapeutics, School of Medicine, National and Kapodistrian University of Athens, 11528 Athens, Greece; eterpos@med.uoa.gr (E.T.); mdimop@med.uoa.gr (M.A.D.)
[4] Hematology Laboratory Blood Bank, School of Medicine, Aretaieion Hospital, National and Kapodistrian University of Athens, 11528 Athens, Greece; mpolitou@med.uoa.gr
[5] Hematology Unit, School of Medicine, Second Department of Internal Medicine, Attikon University General Hospital, National and Kapodistrian University of Athens, 12461 Athens, Greece; vas_pappa@yahoo.com
[6] BMT Unit, Department of Hematology and Lymphomas, Evangelismos General Hospital, 10676 Athens, Greece; marianpagoni@yahoo.com
[7] Department of Transfusion Service and Clinical Hemostasis, "Saint Savvas" Oncology Hospital, 11522 Athens, Greece; egrouzi@otenet.gr
[8] IHU-Méditerranée Infection, Aix-Marseille University, 19-21 Boulevard Jean Moulin, 13005 Marseille, France
* Correspondence: nikiv@pasteur.gr; Tel.: +30-210-647-8875
† These authors contributed equally to this work.

Citation: Labropoulou, S.; Vassilaki, N.; Milona, R.S.; Terpos, E.; Politou, M.; Pappa, V.; Pagoni, M.; Grouzi, E.; Dimopoulos, M.A.; Mentis, A.; et al. Characterizing Kinetics and Avidity of SARS-CoV-2 Antibody Responses in COVID-19 Greek Patients. *Viruses* **2022**, *14*, 758. https://doi.org/10.3390/10.3390/v14040758

Academic Editors: Apostolos Beloukas and Serafeim Chaintoutis

Received: 8 February 2022
Accepted: 31 March 2022
Published: 5 April 2022

Publisher's Note: MDPI stays neutral with regard to jurisdictional claims in published maps and institutional affiliations.

Copyright: © 2022 by the authors. Submitted for possible open access publication under the terms and conditions of the Creative Commons Attribution (CC BY) license (https://creativecommons.org/licenses/by/4.0/).

1. Supplementary Materials and Methods

1.1. Patients

Blood samples were collected in a 5 ml vaccutainer from COVID-19 patients in 3 or 4 time-points over a period of up to 15 months, during their infection and after recovery. All patients had confirmed SARS-CoV-2 infection via a positive RNA nasopharyngeal swab PCR test, the time of which was considered as month zero (T0), and a known date of symptom onset. In total, 35 serum samples were obtained from 10 patients. The serum samples were separated after centrifugation at 3000 rpm for 5 min.

1.2. Serological Assays

Anti-SARS-CoV-2 neutralizing antibodies detection was performed using cPass™ SARS-CoV-2 Neutralizing Antibody Detection Kit (Nanjing GenScript Biotech co., Nanjing, China) according to the manufacturers' protocols. Optical density (OD) of the sample divided by the one of calibrator provided inhibition percentage for which ≥30% indicates the presence of SARS-CoV-2 neutralizing antibodies.

1.3. Statistical analyses

At first, descriptive statistics were calculated. Based on the D'Agostino and Pearson test, replaced by the Shapiro-Wilk test in smaller sample sizes, we verified if data distribution was parametric or non-parametric and subsequently selected the appropriate statistical test for analysis. Analyses of anti-SARS-CoV-2 neutralizing antibody levels in relation to time (four unpaired groups) were performed using Kruskal-Wallis test. Dunn's

correction was used for post hoc analysis of two time-points at a time. Correlation between antibody avidity percentages and antibody neutralizing percentages was determined by Spearman's correlation coefficient (*r*). All statistical analyses were performed using GraphPad (GraphPad Prism version 9.0.0 free trial, GraphPad Software Inc., San Diego, CA, USA) and p<0.05 (two-tailed) was considered statistically significant.

2. Supplementary Figure

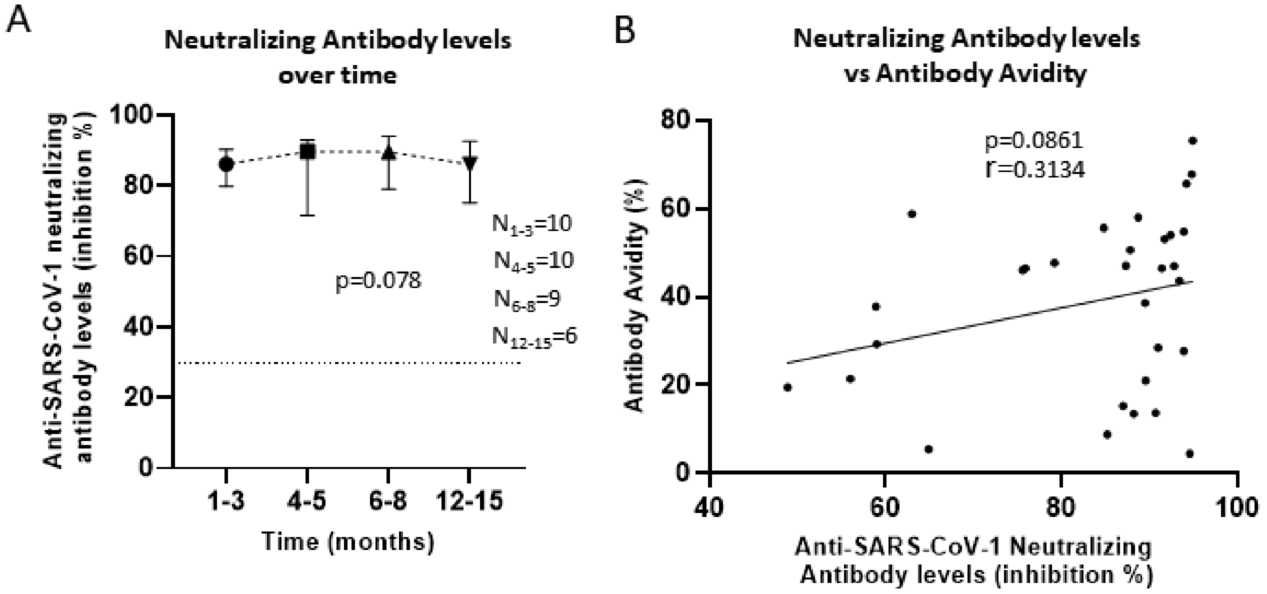

Figure S1. Anti-Spike RBD neutralizing antibody levels in COVID-19 patients over time after infection and correlation with antibody avidity. **(A):** Anti-Spike RBD neutralizing antibody levels were measured, using cPass™ SARS-CoV-2 Neutralizing Antibody Detection Kit, at several time-points up to fifteen months. Points at the graph represent the medians of % inhibition at each time-point and error bars indicate the interquartile range (IQR). Horizontal dashed line represents the cut-off value of the kit used. P value indicated on the graph was calculated using Kruskal-Wallis test for all time-points collectively, while post hoc analysis of two time-points at a time revealed no statistical significance. **(B):** XY scatter plot and linear regression lines of SARS-CoV-2 antibody avidity versus neutralizing antibody levels. Antibody avidity was measured using Euroimmun anti-SARS-CoV-2 ELISA IgG kit and neutralizing antibody levels was measured using cPassTM SARS-CoV-2 Neutralizing Antibody Detection Kit. Pearson's correlation coefficient (r) and p values (p) were calculated.
